# Enzyme-Flavonoid Synergistic Hydrogel: Enables Glucose-Activated Cascade Acidification and Programmed Drug Release for Diabetic Wound Therapy

**DOI:** 10.3390/gels12070652

**Published:** 2026-07-21

**Authors:** Guixi Wang, Sihang Shen, Yichen Tian, Chao Li, Junnan He, Yuzhu Song

**Affiliations:** 1 School of Basic Medical Sciences, Kunming Medical University, Kunming 650500, China; wangguixi2021@163.com; 2 Research Center of Molecular Medicine of Yunnan Province, Faculty of Life Science and Technology, Kunming University of Science and Technology, Kunming 650500, China; ssh011231@163.com (S.S.); 20250094@kust.edu.cn (Y.T.); 20240131@kust.edu.cn (C.L.); 3 Yunnan Key Laboratory of Modern Separation Analysis and Substance Transformation, College of Chemistry and Chemical Engineering, Yunnan Normal University, Kunming 650500, China

**Keywords:** quercetin, glucose/pH-responsive hydrogel, ROS scavenging, immunoregulation, wound healing

## Abstract

Inflammation and oxidative stress induced by high glucose levels constitute essential factors impeding wound healing in diabetes, posing a significant threat to public health. Despite its notable anti-inflammatory and antioxidant potential, the clinical applicability of quercetin is hampered by its hydrophobicity and limited oral bioavailability. To address these issues, the thin-film hydration method was used to encapsulate quercetin into FQ micelles. Subsequently, 3-aminophenylboronic acid-modified oxidized alginate was crosslinked with polyvinyl alcohol, and simultaneously loaded with glucose oxidase (GOX) and FQ micelles, to construct a glucose-activated cascade acidification-triggered controlled-release hydrogel (OSSP@FQ&GOX). The phenylboronic ester bonds in the hydrogel are responsive to glucose and undergo cleavage. GOX-mediated oxidation of glucose produces gluconic acid, resulting in a lower local pH and subsequently triggering FQ micelle release. The released FQ micelles alleviate oxidative stress and exert immunomodulatory effects, while the hydrogel also provides self-healing, and biocompatible properties that facilitate cutaneous regeneration in diabetic mice. Thus, this study highlights the potential of combining GOX with natural products and multi-stimuli-responsive hydrogels for the treatment of chronic diabetic wounds, while also opening new avenues for the development of multifunctional wound dressings.

## 1. Introduction

Diabetes mellitus is a prevalent chronic metabolic disorder characterized by dysregulated glucose homeostasis, affecting approximately 828 million adults worldwide in 2022 [1,2]. Among its diverse complications, diabetic foot ulcers (DFUs) represent the most severe and costly, posing markedly elevated risks of amputation and mortality. Epidemiologically, DFUs affect 19–34% of diabetic patients [3,4], with a 3- to 5-year recurrence rate of ~65%, an overall amputation rate of 20%, and a 5-year mortality rate ranging from 50% to 70% [5,6]. The intractable healing of diabetic ulcers is primarily attributable to disturbances in the local microenvironment. Sustained hyperglycemia leads to substantial accumulation of AGEs within the wound bed, which potentiates excessive reactive oxygen species (ROS) generation and perpetuates chronic inflammation. These pathological perturbations successively compromise extracellular matrix remodeling, granulation tissue formation, and angiogenesis, eventually culminating in healing impairment [7,8,9].

Glucose oxidase (GOX) has been widely used in diabetic wound therapy due to its ability to specifically consume local glucose and generate gluconic acid and hydrogen peroxide (H_2_O_2_) [10]. However, the excessive H_2_O_2_ produced during GOX catalysis can exacerbate oxidative stress and cause secondary tissue damage [11]. In view of the inherent limitation of excessive H_2_O_2_ generation during GOX catalysis, we postulate that co-administration of quercetin, which possesses both antioxidant and anti-inflammatory activities, is of significant importance for relieving GOX-induced oxidative injury. Meanwhile, these factors coordinately regulate the hyperglycemic milieu and mitigate inflammation. Recently, Luo et al. [12] designed a multifunctional nanogel dressing that integrates quercetin and GOX with acid/H_2_O_2_ dual-responsive release capabilities for synergistic treatment of diabetic infected wounds. However, since quercetin serves as a structural component of the gel backbone, its release is governed by the degradation kinetics of the material. Moreover, the hydrogel relies solely on phenylboronate ester crosslinking as its single responsive unit. These two factors may collectively lead to insufficient quercetin release.

Motivated by these challenges, we developed a glucose-activated hydrogel system that enables cascade acidification and programmed drug release, thereby remodeling the wound microenvironment and effectively promoting diabetic wound repair. The smart responsive hydrogel was prepared from 3-aminophenylboronic acid-modified oxidised sodium alginate (OSA) (PBA-OSA) and polyvinyl alcohol (PVA) via phenylboronate ester interactions. GOX and quercetin were incorporated into the hydrogel system. As illustrated in the Graphical Abstract, in the hyperglycemic environment of diabetic wounds, glucose competitively interacts with the phenylboronic acid groups, triggering the disintegration of the hydrogel network. GOX, once released, mediates the oxidation of glucose to gluconic acid and hydrogen peroxide, resulting in a pH drop in the local environment. This acidification further disrupts the Schiff base bonds within the hydrogel, ultimately promoting the release of quercetin micelles. Moreover, quercetin is capable of inhibiting oxidative damage induced by hydrogen peroxide accumulation and exhibits anti-inflammatory activity. Through synergistic components, the OSSP@FQ&GOX hydrogel achieves sequential glucose reduction, oxidative stress modulation, and anti-inflammatory action. By remodeling the microenvironment, it effectively promotes the healing of chronic diabetic wounds, offering new insights for addressing clinical challenges related to chronic wound management.

## 2. Results and Discussion

### 2.1. Characterisation of Micelles FQ

**Figure 1:**
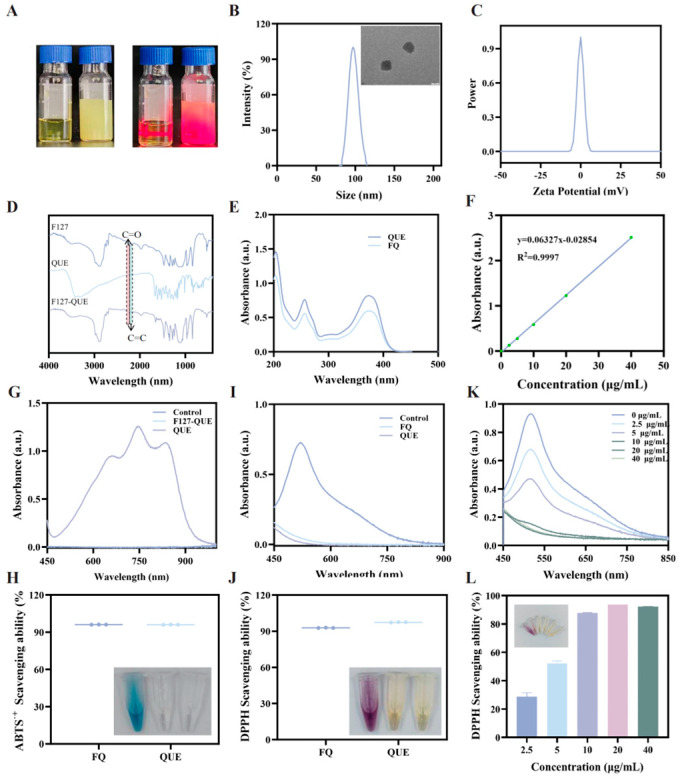
Physicochemical characterization and antioxidant activity of FQ micelles. (**A**) Photographs of FQ micelles in aqueous solution and the Tyndall effect. (**B**) TEM image and hydrodynamic size distribution determined by dynamic light scattering (DLS). (**C**) Zeta potential of FQ micelles. (**D**) FTIR spectra comparison of free quercetin, F127, and drug-loaded micelles (The color-marked features correspond to the characteristic absorption peaks of C=O and C=C). (**E**) UV-Vis absorption spectra comparison of free quercetin and drug-loaded micelles. (**F**) Standard calibration curve of quercetin for calculation of drug loading content and encapsulation efficiency. (**G**–**L**) In vitro scavenging activities of FQ micelles against DPPH• and ABTS^•+^ radicals (*n* = 3).

Quercetin, a natural flavonoid, has attracted considerable attention in diabetic wound therapy due to its antioxidant [13] and anti-inflammatory activities [10,13,14,15]. However, its poor water solubility and low bioavailability severely limit its further clinical application [15,16,17]. We encapsulated quercetin into micelles using the biocompatible triblock copolymer Pluronic^®^ F127 via the film hydration method. The resulting formulation was named FQ. Pluronic^®^ F127 acts as an amphiphilic copolymer [18]. Its hydrophobic segments self-associate in water to form polymeric micelles, which can encapsulate hydrophobic drug molecules within their core [18,19]. This significantly enhances the drug’s apparent solubility and facilitates the delivery of otherwise poorly soluble compounds. As shown in Figure 1A, FQ micelles exhibit uniform transparency, displaying the characteristic scattering phenomenon of micelles known as the Tyndall effect. The strategy significantly enhances the water solubility of quercetin. Transmission electron micrographs showed that FQ micelles were spherical and uniformly dispersed (Figure 1B). The DLS results indicate that the particle size of FQ micelles is about 97.03 nm, and the average size is basically the same as that obtained from the TEM analysis (Figure 1B). Zeta potential, which is the potential difference between the outer layer of the micelles and the solution, is an important measure of electrostatic stabilisation. The average zeta potential of the FQ micelles is close to −0.2 mV, which suggests that the micelles are almost electrically neutral (Figure 1C). Subsequently, we used FTIR and UV-visible absorption spectroscopy for further characterisation. The FQ micelles contained the characteristic F127 absorption peaks in the FTIR spectra, and the C=O absorption peak of quercetin located at 1655 cm^−1^ and the C=C absorption peak at 1599 cm^−1^ were also observed (Figure 1D). Similarly, UV-visible absorption spectroscopy confirms this result, with quercetin and FQ micelles absorbing at 255 and 372 nm wavelengths (Figure 1E). The loading and encapsulation rates of the micelles were calculated from the quercetin standard curve and were 1.95% and 98.28%, respectively. The FQ micelles had the same antioxidant effect as the same concentration of quercetin, which suggests that merely improving the water solubility does not affect its activity (Figure 1G–K and Figure S1). These results demonstrate that FQ micelles were successfully prepared.

### 2.2. Characterization of Hydrogels

The complex and dynamic nature of diabetic wound healing demands therapeutic platforms capable of not only delivering drugs but also actively remodeling the local microenvironment—a requirement that hydrogels, with their rationally designed cross-linked networks, are uniquely positioned to fulfill. Stimuli-responsive hydrogels can undergo reversible physicochemical transitions when exposed to exogenous or endogenous triggers, such as pH, temperature, glucose, or reactive oxygen species [20]. This unique property makes them particularly suitable for on-demand and spatiotemporally controlled drug delivery [20,21]. In the specific setting of diabetic wounds, hydrogels engineered with dynamic covalent bonds, including Schiff base linkages and boronate ester bonds, offer a distinct therapeutic edge, as they sense pathological cues characteristic of the diabetic milieu, namely persistent hyperglycemia, local acidosis, and excessive oxidative stress, and translate these signals into triggered degradation and programmable cargo release [22]. However, existing single-stimulus response strategies often result in insufficient drug release due to limited responsiveness, making it difficult to adapt to the complex healing cycle of diabetic wounds [23]. To circumvent these shortcomings, we rationally designed a dual-responsive hydrogel system that exploits both Schiff base and aminophenylboronic acid chemistry to achieve glucose/pH cascade responsiveness. This platform not only enables the co-delivery of quercetin and GOX but also ensures accelerated quercetin liberation specifically under hyperglycemic and acidic microenvironments, leveraging its intrinsic antioxidant and anti-inflammatory activities to reprogram the diabetic wound milieu.

**Figure 2:**
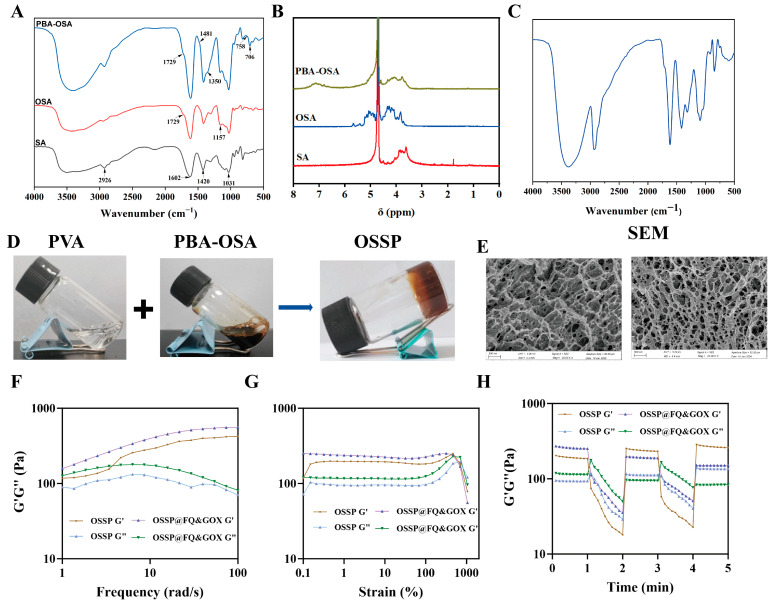
Structural and rheological characterization of hydrogels. (**A**) FTIR spectra comparison of sodium alginate (SA), oxidized SA (OSA), and phenylboronic acid-grafted OSA (PBA-OSA). (**B**) ^1^H NMR spectra of SA, OSA, and PBA-OSA, confirming their chemical structures. (**C**) FTIR spectrum of the crosslinked composite hydrogel. (**D**) Schematic illustration of the hydrogel preparation process. (**E**) Scanning electron microscopy (SEM) image of the freeze-dried hydrogel, revealing its internal porous microstructure. (**F**–**H**) Rheological properties of the hydrogels, including frequency sweep (**F**), amplitude sweep (**G**), and step-strain recovery test (**H**).

Sodium alginate, a representative natural polysaccharide, forms the basis of its hydrogels [24,25]. With excellent biocompatibility, degradability, renewability and low cost advantages, this type of hydrogel has become one of the ideal materials in the biomedical field [26]. OSA was obtained by oxidation reaction of sodium alginate by sodium periodate. Subsequently, OSA was obtained by grafting with 3-aminophenylboronic acid (PBA) using Schiff base reaction to obtain PBA-OSA. Figure S2 illustrates the UV-Vis spectra recorded for SA, OSA, and PBA-OSA. While PBA exhibits characteristic absorption bands at 232 nm and 296 nm, which are ascribed to its benzene ring structure, OSA does not exhibit UV-visible absorption peaks in the range of 220–400 nm, but PBA-OSA has absorption peaks at 246 nm and 305 nm, which is similar to that of PBA with the characteristic benzene ring absorption peaks. Because the amino group forms a Schiff base bond with the benzene ring creating a π-π conjugation effect resulting in a shift of the PBA-OSA absorption peaks (Figure 2A). The grafting ratio of 3-PBA was 39.3%. OSA appeared as a C=O stretching vibration peak of the carbonyl group at 1729 cm^−1^ for PBA-OSA. The characteristic peaks of the interstitial substitution of the benzene ring appeared at 706 and 758 cm^−1^ for PBA-OSA, 1350 cm^−1^ for the B-O stretching vibration absorption peaks on phenylboronic acid at 1350 cm^−1^ and a phenyl ring backbone stretching vibration peak at 1481 cm^−1^. In addition, the structures of OSA and PBA-OSA were further determined using ^1^H NMR. The hemiacetal derived from the aldehyde group with the neighbouring hydroxyl group were observed at δ = 5.16, 5.47 and 5.66 for OSA, as shown in Figure 2B. Comparing with OSA, the characteristic peaks belonging to the proton peaks of benzene ring appear in the δ = 7.3–7.6 of the PBA-OSA spectrum. These results indicated that PBA-OSA was successfully synthesized.

The OSSP hydrogel was readily prepared by simply mixing PBA-OSA and PVA at room temperature for 15 s, without the need for crosslinkers or heating, which is attributed to the reversible boronate ester interactions between the PBA moieties and the diol units of PVA. The formation mechanism of the hydrogel was verified by FT-IR spectroscopy (Figure 2C). Comparison of the FT-IR spectra of PBA-OSA and the hydrogel revealed that the characteristic absorption peak at 1350 cm^−1^, assigned to the B-O stretching vibration in PBA-OSA, shifted to 1318 cm^−1^ upon gelation, accompanied by a more symmetric peak shape. This red shift is ascribed to the change in electron cloud density around the boron atom resulting from the formation of B-O-C bonds. The precursor solutions (PBA-OSA and PVA) were flowable, whereas the resulting hydrogel lost its flowability after mixing (Figure 2D). Scanning electron microscopy (SEM) images showed that both OSSP and OSSP@FQ&GOX possessed a three-dimensional porous network structure (Figure 2E), which resembles the natural extracellular matrix and is favorable for cell adhesion and proliferation. The porosity of the hydrogels was evaluated using the ethanol displacement method, and the porosities of the OSSP and OSSP@FQ&GOX hydrogels were determined to be 84.59% and 80.92%, respectively.

### 2.3. The Rheological Properties, Injectability, Self-Healing Capability, Swelling Behavior, and Degradation Performance of the Hydrogels

**Figure 3:**
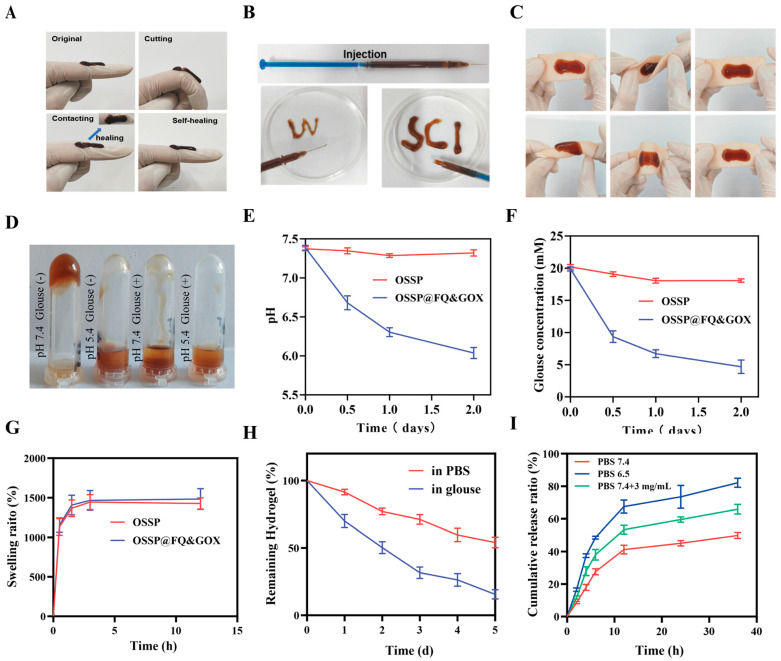
Injectable, adhesive, stimuli-responsive (pH/glucose), and in vitro performance of hydrogels. (**A**) Self-healing properties of the hydrogel. (**B**) Injectability of the hydrogel. (**C**) Adhesion properties of the hydrogel. (**D**) pH- and glucose-responsive behavior of the hydrogel. (**E**) Time-dependent pH changes in DMEM culture medium. (**F**) Time-dependent glucose concentration changes in DMEM culture medium. (**G**) Swelling ratios of OSSP and OSSP@FQ&GOX hydrogels. (**H**) Degradation rates of OSSP@FQ&GOX in PBS and high-glucose solutions. (**I**) Cumulative release profiles of quercetin from OSSP@FQ&GOX hydrogels at pH 7.4, pH 6.5, and under high-glucose conditions.

Dynamic covalent cross-linking endows hydrogels with exceptional injectability and self-healing properties. Injectability enables precise filling and conforming to irregular wound surfaces, whilst self-healing capability ensures rapid restoration of structural integrity following mechanical stress, thereby extending the functional lifespan of the dressing. As illustrated in Figure 3B, when loaded into a 1 mL syringe, the hydrogel was continuously and smoothly extruded through the needle tip under constant thrust, successfully forming the characters “W” and “SCI”. This visually demonstrates its excellent injectability and shape adaptability. Self-healing performance was validated through cut-reconnection experiments: hydrogel coated on a finger surface was severed during bending, and upon contact between the cut ends, it autonomously repaired within a short timeframe, restoring its complete structure (Figure 3A). Furthermore, pig skin adhesion tests demonstrated the hydrogel’s robust tissue adhesion. Even after repeated bending and twisting of the pig skin, the hydrogel remained firmly attached without peeling or detachment (Figure 3C). We quantitatively evaluated the interfacial adhesion performance of the OSSP and OSSP@FQ&GOX hydrogels using lap-shear strength measurements. The peak loads of the OSSP and OSSP@FQ&GOX hydrogels were recorded as 0.702 N and 0.741 N, respectively, corresponding to a peak shear stress of approximately 0.001 MPa in both cases.

Using a rheometer, we performed dynamic oscillatory frequency and strain scans to probe the mechanical performance of the hydrogel. As shown in Figure 2F, the frequency scan results confirmed the successful formation of the hydrogel. Throughout the tested frequencies (1–10 Hz), a consistent pattern of G′ > G″ was observed. Further strain scanning revealed (Figure 2G) that as strain increased, the hydrogel’s response transitioned from G′ > G″ to G′ < G″, indicating a shift in behaviour from solid-like to fluid-like. This intuitively demonstrates the hydrogel’s fluidity and capacity to fill irregular wounds. Additionally, the hydrogel’s self-healing performance was evaluated via continuous step strain testing. Results are shown in Figure 2H: upon application of a large strain of 200%, the network undergoes temporary dissociation (G″ > G′), exhibiting liquid-like behaviour; once strain is reduced to 1%, rapid reorganization based on dynamic covalent bonds immediately restores G′ and G″ to their initial values, demonstrating excellent self-healing performance.

An ideal wound dressing should possess both good swelling and degradation properties [27]. The swelling property facilitates the absorption of wound exudate, while the degradation property not only promotes drug release but also shortens the dressing change interval [28,29,30]. As shown in Figure 2G, both OSSP and OSSP@FQ&GOX hydrogels exhibited excellent swelling behavior, with values exceeding 1400%. On this basis, we further evaluated the degradation performance of the OSSP@FQ&GOX hydrogel. As depicted in Figure 3H, the hydrogel showed favorable degradation behavior, and its degradation rate was significantly accelerated in a high-glucose solution.

### 2.4. The pH and Glucose Responsiveness and Drug Release of the Hydrogel

The OSSP@FQ&GOX hydrogel incorporates dynamic/reversible Schiff base bonds and borate ester bonds, exhibiting responsive behavior under low pH and high glucose conditions. As clearly observed in the macroscopic images (Figure 3D), the hydrogel network collapses and transitions into a flowable state under low pH and high glucose conditions. High glucose, a hallmark feature of diabetic wounds, serves as a trigger to initiate network collapse and promote drug release. GOX within the OSSP@FQ&GOX hydrogel can oxidize glucose to produce gluconic acid, thereby effectively creating an acidic pH environment that accelerates network disintegration. As shown in Figure 3E, after mixing the OSSP@FQ&GOX hydrogel with DMEM culture medium, the pH decreased from 7.38 to 6.03. Meanwhile, the glucose concentration in the solution was reduced from 19.0 mM to 4.68 mM upon treatment with the OSSP@FQ&GOX hydrogel (Figure 3F). Subsequently, we investigated the release behavior of quercetin and GOX from the OSSP@FQ&GOX hydrogel. As shown in Figure 3I, the cumulative release of quercetin in PBS (pH 7.4) reached 49.83% within 36 h. When the pH was lowered or the glucose concentration was increased, the release rate was significantly accelerated, with the cumulative release increasing to 82.13% and 65.87% at 36 h. This release behavior is attributed to the pH responsiveness of the Schiff base linkages and the glucose responsiveness of the phenylboronate ester groups [31].

### 2.5. Biocompatibility of Hydrogels

**Figure 4:**
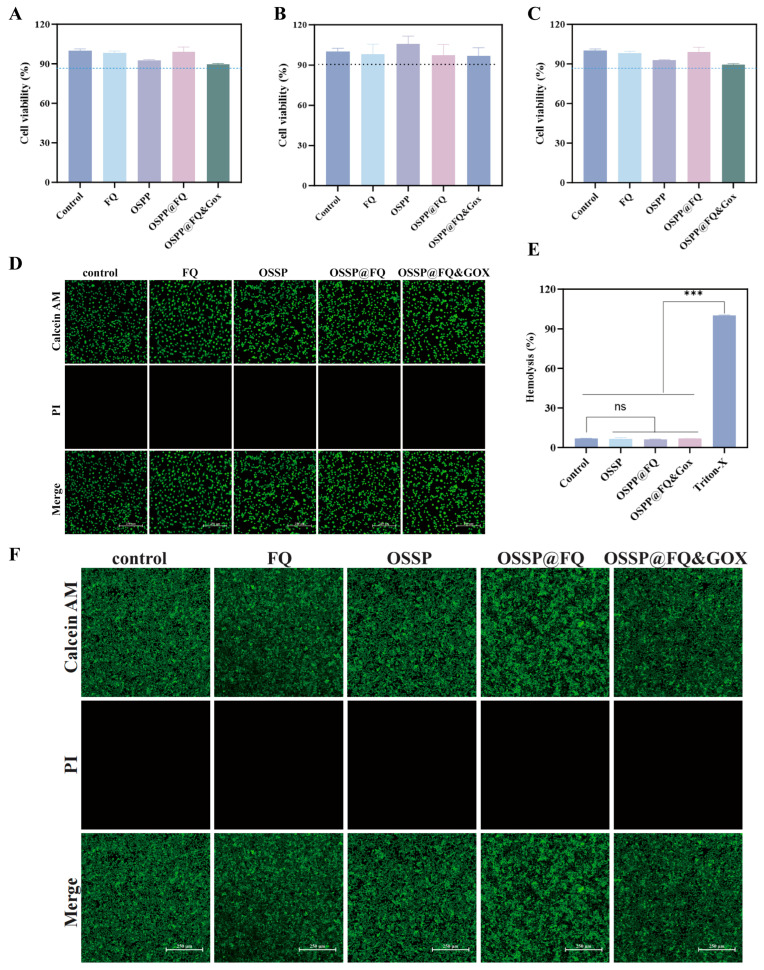
Biocompatibility assessment of the hydrogels. (**A**–**C**) CCK-8 assays for evaluating the cytotoxicity of hydrogels against HUVECs, L929, and RAW264.7 cells (*n* = 3) *** *p* < 0.001, ns, not significant. (**D**,**E**) Calcein-AM/PI live/dead cell staining for assessing hydrogel toxicity toward HUVECs (**D**) and RAW264.7 cells (**E**) (*n* = 3). (**F**) Hemocompatibility evaluation of the hydrogels (*n* = 3).

Biocompatibility serves as a fundamental prerequisite for any material designed for biomedical use [32,33,34]. No obvious cytotoxicity was observed for any of the tested formulations (FQ micelles, OSSP, OSSP@FQ, and OSSP@FQ&GOX), as all groups maintained cell viabilities above 85% in HUVECs, L929, and RAW264.7 cells compared with the control (Figure 4A,C). Consistently, Calcein-AM/PI double staining yielded intense green fluorescence and minimal red fluorescence, corroborating the satisfactory biocompatibility of the hydrogel materials (Figure 4D,F). As shown in Figure 3E and Figure S5, the hemolysis assay demonstrated that none of the tested hydrogels (OSSP, OSSP@FQ, and OSSP@FQ&GOX) caused detectable hemolysis in mouse red blood cells compared with the PBS control. Quantitative measurements revealed no significant difference in hemolysis rates between the hydrogel groups and the blank control, meeting the basic hemocompatibility standards for biomaterials intended for clinical use. Following two weeks of subcutaneous implantation, histopathological evaluation of major organs (heart, liver, spleen, lungs, and kidneys) was performed (Figure S8). Hematoxylin and eosin (H&E) staining indicated that neither the FQ micelle nor the hydrogel groups induced any overt histopathological alterations in the examined tissues. These results collectively attest to the favorable biocompatibility of the hydrogel, thereby supporting its application in cell-based assays and paving the way for future in vivo wound healing studies.

### 2.6. Antioxidant Activity of the Hydrogels

**Figure 5:**
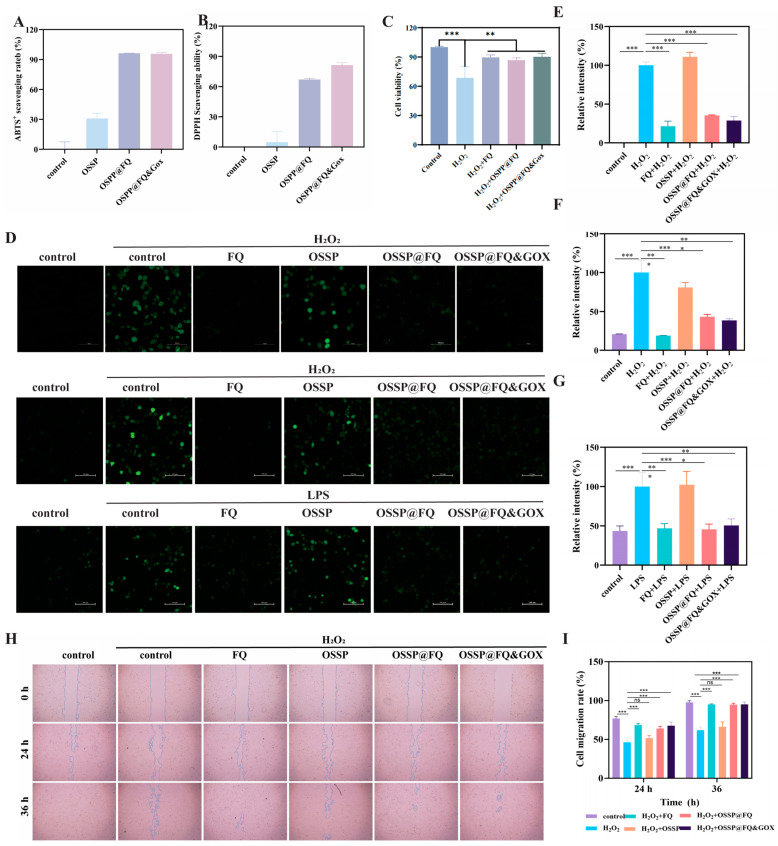
In vitro antioxidant properties of the hydrogels. (**A**,**B**) Corresponding radical scavenging activities of the hydrogels against ABTS^+^• (**A**) and DPPH• (**B**). (**C**) Protective effects of hydrogel extracts or FQ micelles against H_2_O_2_-induced oxidative damage in HUVECs. (**D**) Representative fluorescence images of intracellular ROS levels in HUVECs, L929, and RAW264.7 cells after treatment with hydrogel extracts or FQ micelles (scale bar: 100 μm). (**E**–**G**) Quantitative analysis of ROS fluorescence intensity in HUVECs (**E**), L929 (**F**), and RAW264.7 cells (**G**). (**H**,**I**) Effects of hydrogel extracts or FQ micelles on the migration ability of HUVECs under H_2_O_2_-induced oxidative stress conditions, shown as wound-healing assay images (**H**) and quantitative analysis of migration rates (**I**). * *p* < 0.05, ** *p* < 0.01, *** *p* < 0.001 (*n* = 3), ns, not significant.

Redox imbalance is a critical barrier to diabetic wound repair [35,36]. Excessive ROS not only inflict direct oxidative damage (lipid peroxidation, DNA lesions, mitochondrial dysfunction) culminating in cell death, but also perpetuate inflammatory signaling and compromise fibroblast/endothelial cell activity, thereby hindering granulation and angiogenesis [37]; additionally, the H_2_O_2_ produced by GOX-catalyzed glucose oxidation further exacerbates oxidative injury and causes GOX inactivation. The parent OSSP hydrogel demonstrated suboptimal antioxidant performance (Figure 5A,B), yet the incorporation of quercetin endowed OSSP@FQ and OSSP@FQ&GOX with pronounced scavenging capacity, eradicating >65% of DPPH and ABTS^•+^radicals. To probe the cytoprotective efficacy of OSSP@FQ&GOX against oxidative insult, HUVECs were challenged with a high dose of H_2_O_2_ to recapitulate a ROS-enriched milieu. Exposure to H_2_O_2_ provoked a sharp decline in cell viability (<68.5%), whereas pretreatment with FQ micelles, OSSP@FQ, or OSSP@FQ&GOX significantly restored viability to 89.5%, 86.5%, and 90%, respectively (Figure 5C). To dissect the mechanistic basis, intracellular ROS accumulation was assessed by DCFH-DA oxidation (Figure 5D,E). H_2_O_2_-challenged cells displayed bright green fluorescence, reflecting a surge in ROS generation. Conversely, cells pretreated with hydrogel extracts of OSSP@FQ or OSSP@FQ&GOX exhibited conspicuously attenuated fluorescence. The unmodified OSSP hydrogel, however, exerted negligible ROS-scavenging action. Additionally, a scratch assay was employed to evaluate the influence of oxidative stress on cell motility. Figure 5H illustrates that H_2_O_2_ profoundly suppressed migratory ability, which was effectively rescued by hydrogel extracts of OSSP@FQ or OSSP@FQ&GOX administration. Considering the reciprocal reinforcement between chronic inflammation and oxidative stress in diabetic pathology, we also interrogated whether OSSP@FQ&GOX could curtail LPS-evoked ROS production (Figure 5D,G). The data confirmed its capacity to abate intracellular ROS in LPS-treated cells. Overall, these results substantiate that OSSP@FQ and OSSP@FQ&GOX hydrogels facilitate cellular proliferation and migration through ROS elimination and oxidative injury mitigation, laying a robust groundwork for their translational potential in tissue regeneration.

### 2.7. Anti-Inflammatory Activity and Macrophage Reprogramming by Hydrogels

**Figure 6:**
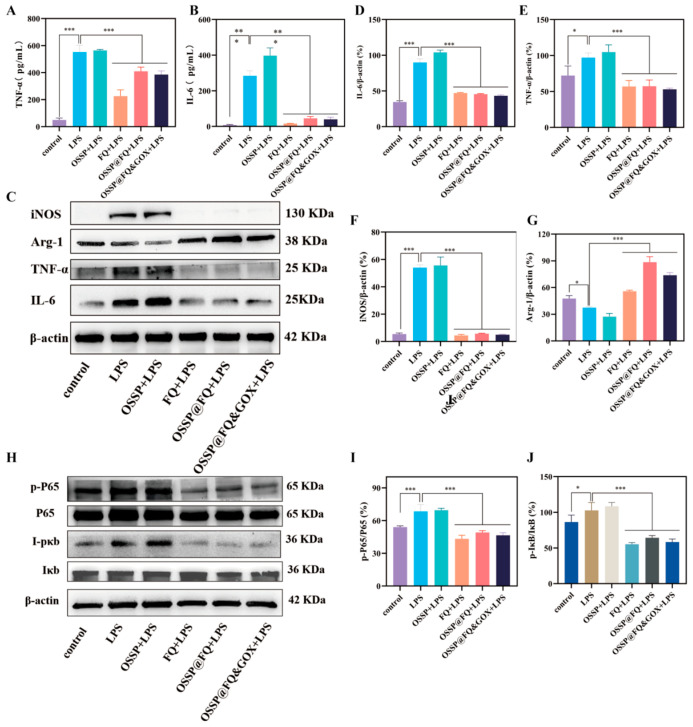
Immunomodulatory Activity of the Hydrogels in vitro.(**A**,**B**) The levels of pro-inflammatory factors in Raw 264.7 cell supernatants were measured after stimulation with 200 ng/mL LPS. (**C**) Protein expression levels assessed by Western blot. (**D**–**G**) Densitometric analysis of the relative protein levels for IL-6, TNF-α, iNOS, and Arg-1 respectively. (**H**) Protein expression levels measured by Western blot. (**I**,**J**): Statistical analysis of P65 and IκB protein phosphorylation. * *p* < 0.05, ** *p* < 0.01, *** *p* < 0.001 (*n* = 3).

Macrophages serve as key arbiters of inflammation resolution and tissue repair in wound healing [38,39]. In diabetic wounds, the early inflammatory milieu is dominated by M1-type macrophages, which release high levels of tumour necrosis factor-α (TNF-α) and interleukin-6 (IL-6). Sustained M1 accumulation fuels persistent inflammation, inflicts bystander tissue damage, and curtails the function of regenerative cells, thereby stalling wound closure. Conversely, M2 macrophages are instrumental in quenching inflammation and promoting regenerative processes. Consequently, inducing a functional shift from M1 to M2 polarization has emerged as a rational intervention to subvert chronic inflammation and reinstate the healing cascade. Compared with the LPS group, OSSP@FQ&GOX treatment markedly reduced the expression of both TNF-α and IL-6 at the cellular level. Western blot results further confirmed this finding (Figure 6C–E). Given that inducible nitric oxide synthase (iNOS) and Arginase-1 (Arg-1) are key markers of M1 and M2 macrophage polarization, respectively, the downregulation of iNOS and upregulation of Arg-1 observed after treatment with the hydrogel extracts of OSSP@FQ&GOX extract in this study suggest that this system may promote the polarization shift of macrophages from the pro-inflammatory M1 phenotype toward the reparative M2 phenotype. Further detection of CD86 (a marker of M1 macrophages) showed that OSSP@FQ&GOX inhibited its expression (Figure S6). The flow cytometry results demonstrated that the OSSP@FQ&GOX hydrogel significantly lowered the proportion of CD86-positive cells, which were mutually corroborated by the Western blot analyses (Figure S7). The anti-inflammatory effect of quercetin is closely associated with the NF-κB signaling pathway, primarily through inhibition of IκB kinase (IKK) phosphorylation, which prevents IκBα degradation and subsequent nuclear translocation of NF-κB, thereby reducing the transcriptional expression of downstream pro-inflammatory cytokines. To investigate whether this pathway mediates the anti-inflammatory action of the hydrogel system, we examined the protein levels of phosphorylated p65 (p-p65) and phosphorylated IκB (p-IκB) in LPS-stimulated Raw 264.7 cells by Western blotting. As shown in Figure 6H–J, LPS challenge markedly elevated p-p65 and p-IκB levels, confirming activation of the NF-κB pathway. In contrast, treatment with the hydrogel extracts of OSSP@FQ and OSSP@FQ&GOX or FQ micelles significantly reduced both p-p65 and p-IκB expression. Collectively, these results unequivocally demonstrate that the anti-inflammatory activity of the hydrogel originates from the quercetin payload, which negatively regulates the NF-κB axis.

### 2.8. Evaluation of Diabetic Wound Healing Ability of OSSP@FQ&GOX In Vivo

**Figure 7:**
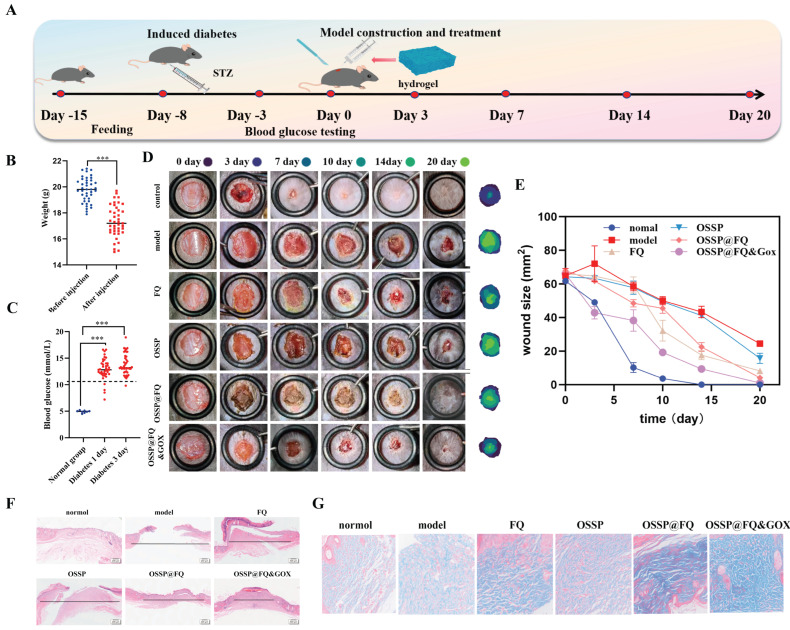
In vivo wound healing performance of hydrogels in diabetic mice over a 20-day treatment period. (**A**) Schematic illustration of the establishment of a full-thickness skin wound model in STZ-induced diabetic mice and the subsequent treatment regimen. (**B**) Body weight changes of diabetic mice after STZ injection. (**C**) Blood glucose levels of diabetic mice after STZ injection. (**D**) Representative photographs of wound closure in diabetic mice at indicated time points after treatment with different formulations. (**E**) Quantitative analysis of wound healing rates over the 20-day treatment period. (**F**,**G**) H&E and Masson’s trichrome staining of wound tissues after 20 days of treatment, showing tissue regeneration and collagen deposition. *** *p* < 0.001 (*n* = 3).

Previous in vitro results demonstrated that the OSSP@FQ&GOX hydrogel exhibits excellent biocompatibility, antioxidant activity, and anti-inflammatory properties, positioning it as a promising candidate for diabetic wound treatment. As depicted in Figure 7A, a full-thickness skin wound model was generated in type I diabetic mice to assess the in vivo therapeutic performance of the OSSP@FQ&GOX hydrogel. During the induction of diabetes, the model mice exhibited significant weight loss (Figure 7B), accompanied by polyphagia and polyuria, which are typical diabetic symptoms. Macroscopic evaluation of wound healing revealed that the wound closure rates were significantly higher in normal mice than in diabetic mice. Among the diabetic models, all treatment groups (FQ, OSSP, OSSP@FQ, and OSSP@FQ&GOX) showed markedly improved wound healing rates compared with the model group (Figure 7D). Notably, the OSSP@FQ&GOX group displayed the most favorable healing kinetics, achieving the fastest wound closure. By day 20, the diabetic model group reached only 50% wound closure, whereas the OSSP@FQ&GOX group achieved complete wound healing (Figure 7E). Furthermore, the superior efficacy of the OSSP@FQ&GOX group over the OSSP@FQ group was attributed to the incorporation of GOX, which not only alleviates glucose-induced cellular damage but also facilitates the release of quercetin, thereby exerting synergistic anti-inflammatory and antioxidant effects.

### 2.9. Histopathological Evaluation of Mice Skin

To evaluate the therapeutic effects of the hydrogel groups on diabetic wounds, we performed histopathological evaluation of the skin tissue. Wound healing in normal mice has approached that of normal tissue. Compared to diabetic mice in all groups except normal mice, treatment with OSSP@FQ&GOX hydrogel markedly reduced wound size, accompanied by the formation of skin appendages such as hair follicles and sweat glands within the new granulation tissue (Figure 7F). During the healing process, skin epithelialisation exhibited a pattern of initial thickening followed by regression. The epidermis in the OSSP@FQ&GOX hydrogel group exhibited the thinnest layer, indicating that re-epithelialisation had undergone regression. Masson’s trichrome staining was further employed to evaluate collagen deposition across different treatment groups (Figure 7G). Collagen fibres in the OSSP@FQ&GOX group exhibited a wavy morphology with an ordered orientation and reticular spatial structure, demonstrating more mature characteristics. In contrast, collagen fibres in the other groups showed relatively underdeveloped organisation, appearing more dispersed and structurally lax.

**Figure 8:**
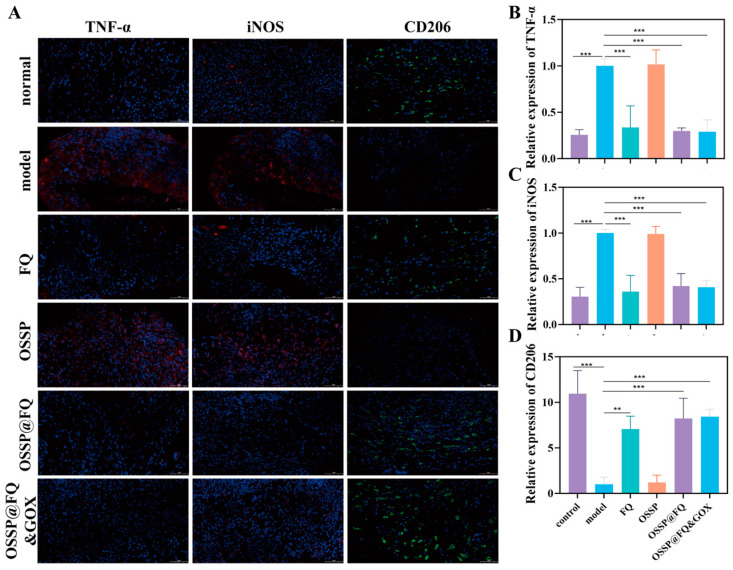
Immunofluorescence analysis of inflammatory and regenerative markers in wound tissues. (**A**) Representative immunofluorescence staining images of TNF-α (pro-inflammatory, red), iNOS (M1 macrophage marker, red), and CD206 (M2 macrophage marker, green) in wound tissues after 20 days of treatment. (**B**–**D**) Quantitative analysis of relative fluorescence intensity for TNF-α (**B**), iNOS (**C**), and CD206 (**D**) in each treatment group. ** *p* < 0.01, *** *p* < 0.001.

Immunofluorescence staining further demonstrated that, in the diabetic model group, wound tissues exhibited significant upregulation of the pro-inflammatory cytokine TNF-α and the M1-associated marker iNOS, alongside a concurrent decrease in the M2 marker CD206, suggesting a state of sustained inflammation within the wound site (Figure 8). In contrast, treatment with OSSP@FQ and, more notably, OSSP@FQ&GOX effectively dampened the inflammatory response through modulation of macrophage polarization. Diabetic chronic wounds frequently exhibit impaired angiogenesis, which results in inadequate nutrient delivery and metabolic waste buildup, thus markedly retarding wound healing. CD31, a specific marker of endothelial cells, is an indicator of neovascularization [40]. As shown in Figure S9, the CD31 immunofluorescence intensity in the OSSP@FQ&GOX and OSSP@FQ group was significantly higher than that in the diabetic group, indicating that this complex promotes wound neovascularization. These results demonstrate that the OSPP@FQ&GOX hydrogel can significantly shorten the inflammatory phase of diabetic wounds by promoting M2 polarization and promoting angiogenesis, thereby accelerating diabetic wound healing.

## 3. Conclusions

Herein, we report a glucose-activated cascade-responsive hydrogel system (denoted as OSSP@FQ&GOX) designed for the treatment of diabetic foot ulcers. The hydrogel is constructed with a dynamic phenylboronate ester network that co-encapsulates GOX as a glucose-lowering initiator and quercetin as an antioxidant and anti-inflammatory agent, enabling degradation-triggered and on-demand drug release. Under hyperglycemic conditions, competitive binding of glucose to the phenylboronic acid moieties disrupts the crosslinked network, facilitating the liberation of entrapped GOX. Subsequently, GOX catalyzes the oxidation of glucose to gluconic acid, resulting in a localized decrease in pH. The acidic microenvironment further accelerates hydrogel degradation, leading to the release of quercetin-loaded micelles, which effectively scavenge ROS and downregulate pro-inflammatory cytokine expression. Notably, the co-delivery system also alleviates GOX-induced oxidative stress. Collectively, the OSSP@FQ&GOX hydrogel not only reduces local glucose levels but also modulates oxidative stress and inflammation, thereby remodeling the hostile diabetic wound milieu and expediting tissue repair. This study presents a promising paradigm for microenvironment-adaptive therapy and offers novel perspectives for the clinical management of diabetic foot ulcers. Despite these promising results, we acknowledge that several aspects warrant further investigation. These include real-time in vivo monitoring of microenvironmental parameters, systematic long-term safety evaluation, validation in infection-complicated models, and assessment in more clinically relevant chronic wound models. Future studies addressing these aspects will be essential to fully establish the translational potential of this platform.

## 4. Materials and Methods

### 4.1. Materials

Quercetin, 3-aminophenylboronic acid, and oxidized sodium alginate were purchased from Macklin (Shanghai, China). Pluronic^®^ F127 and PVA were supplied by Aladdin (Shanghai, China). Antibodies against Arg-1, iNOS, TNF-α, and IL-6 were acquired from Abcam (Shanghai, China). CD86 and CD206 antibodies were provided by WANLEIBIO (Shenyang, China). IκB and phosphorylated-IκB (p-IκB) antibodies were sourced from Abmart (Shanghai, China). p65, phosphorylated-p65 (p-p65) antibodies, and the ROS assay kit were commercially sourced from Servicebio (Wuhan, China). Raw 264.7 cells used in this study were purchased from Solarbio (Beijing, China). The HUVECs (human umbilical vein endothelial cells) and L929 cells are stock cells maintained in our laboratory.

### 4.2. Animals

All animals used in this experiment were supplied by Spefford (Beijing, China). The study was approved by Kunming University of Science and Technology Experimental Animal Ethics Committee (approval No. PZWH-KUST-202506020048-1, dated 15 June 2025).

### 4.3. Preparation of FQ

Polymeric micelles encapsulating quercetin were prepared using the thin film hydration method [41]. Briefly, 400 mg of F127 was added to 10 mL of ethanol and dissolved at 30–40 °C under magnetic stirring. Then 8 mg of quercetin was weighed and dissolved in 2 mL of ethanol solution and added dropwise to the above polymer solution. The quercetin was allowed to fully interact with F127 by using magnetic stirring for 2 h. The quercetin was dissolved in 10 mL of ethanol. The ethanol solvent was removed using a rotary evaporator at a temperature of 40 °C, and the reaction flask was dried under vacuum for 12 h. 20 mL of deionised water was added to the reaction flask, and the quercetin-encapsulated micellar solution was obtained by magnetic stirring at 40 °C for 40 min. The FQ micelles were isolated by centrifugation (4000 rpm, 10 min), purified via filtration (0.45 μm membrane), and freeze-dried into a yellow powder. The final product was stored at 4 °C protected from light.

### 4.4. Micellar FQ Characterisation

The morphological characteristics of micelles FQ were analysed using transmission electron microscopy. The particle size and zeta potential of the micelles were analysed using ZetaPALS^®^ particle size analyser (brookhaven, Holtsville, NY, USA). The presence of quercetin was demonstrated using FTIR spectroscopy and UV spectrophotometry (shimadzu, Kyoto, Japan).

### 4.5. Drug Loading and Encapsulation Rates of FQ Micelles

The concentration of quercetin in FQ micelles was determined using a UV-visible spectrophotometer at 345 nm. Briefly, a standard solution was first prepared by weighing 1 mg of quercetin dissolved in 1 mL of ethanol solution, and different concentrations were prepared to determine its absorbance at 345 nm to obtain a standard curve. Then 1 mg of micellar FQ lyophilised powder was added to 1 mL of H_2_O and dissolved.

FQ micelles were weighed and dissolved in 1 mL of solution, and the content of quercetin in the micelles was quantified by the standard curve. Entrapment efficiency and loading efficiency were calculated according to the following formula.

$$\mathrm{Entrapment}\;\mathrm{efficiency}\;(\%)=\frac{m_{\left(\mathrm{que}\right)}}{m_{\left(\mathrm{initial}\right)}}\times 100\%$$

$$\mathrm{Loading}\;\mathrm{efficiency}\;(\%)=\frac{m_{\left(\mathrm{que}\right)}}{m_{\left(\mathrm{FQ}\right)}}\times 100\%$$

### 4.6. Synthesis and Characterization of PBA-OSA

The synthesis of OSA was conducted with reference to a reported method with slight modifications [42]. The specific procedure is as follows: 4.0 g of sodium alginate (SA) was dissolved with stirring in 266.7 mL of deionized water (pH 5.0). Subsequently, 2.14 g of sodium periodate (NaIO_4_) was added to the solution, and the reaction was allowed to proceed at room temperature for 18 h in the absence of light. Upon completion, 4 mL of ethylene glycol was added to quench any unreacted periodate. The reaction mixture was purified via dialysis against deionized water using a 3500 Da MWCO membrane (solarbio, China) for 3 days for purification, followed by freeze-drying to obtain OSA. The oxidation degree of OSA was measured by hydroxylamine hydrochloride titration: 0.1 g of OSA was mixed with methyl orange/hydroxylamine hydrochloride reagent and titrated with 0.1 M NaOH until the solution changed color from red to yellow, and the oxidation degree was calculated to be 49.05%.

For the preparation of 3-aminophenylboronic acid (3-APBA) modified OSA, the as-synthesized OSA was dissolved in deionized water. 3-APBA was then added, and the modification was carried out via a Schiff base reaction under continuous stirring at room temperature for 18 h. After the reaction, the mixture was similarly dialyzed for 3 days and freeze-dried to collect the final product. The chemical structures of the resulting products were characterized by ^1^H NMR spectroscopy, FT-IR spectroscopy, and UV-Vis spectrophotometry.

### 4.7. Preparation of Hydrogels

The OSSP hydrogel was synthesized through a cross-linking reaction between OSA-PBA and PVA. Briefly, a PBA-OSA solution (3% *w*/*v*) was mixed with a PVA solution (6% *w*/*v*) at a 1:1 volume ratio to form the OSSP hydrogel. Subsequently, the OSSP@FQ&GOX composite hydrogel was fabricated by adding GOX solution (0.25 mg/mL) and an FQ micellar solution (5 mg/mL) into the mixture under continuous stirring.

### 4.8. Rheological Properties and the Stimuli-Responsive (pH/Glucose) Properties of the Hydrogels

Rheological testing of the hydrogel was conducted using a rheometer to measure its storage modulus and loss modulus (G″) at 37 °C [43]. Frequency sweep tests were performed at a fixed strain of 1%, covering an angular frequency range from 1 to 100 rad/s; strain sweep tests were completed at a fixed frequency of 1 Hz, with the strain amplitude incrementally increased from 0.01% to 500%. Furthermore, to evaluate the material’s self-healing properties, continuous step strain tests were conducted. At a constant frequency of 1.0 rad/s, alternating strain scans were performed on the hydrogel at 1% and 200% strain values, recording changes in the sample’s G′ and G″ values.

pH and glucose-response properties were used as the Experiment Reference [44]. The OSSP@FQ&GOX hydrogel (1 mL), prepared as described above, was immersed in 10 mL of PBS containing specific glucose concentrations and pH values. At predetermined time intervals, 1 mL of the release medium was withdrawn and replaced with an equal volume of fresh PBS to maintain a constant total volume. The amount of quercetin released was determined by high-performance liquid chromatography.

### 4.9. Cell Culture and Cytotoxicity Assay

The CCK-8 assay and live/dead cell staining were employed to evaluate the cytotoxicity of the prepared hydrogels. Briefly, HUVECs, L929, and Raw 264.7 cells were prepared as single-cell suspensions and seeded into plates for overnight culture. After 24 h of treatment with hydrogel extracts, the cells were subjected to the indicated assays, all of which were conducted in strict accordance with the manufacturer’s recommended procedures.

### 4.10. Hemolytic Activity of the Hydrogels

A standard hemolysis assay was employed to evaluate hemocompatibility [45,46]. Briefly, mouse whole blood was washed multiple times with normal saline (0.9% NaCl) until the supernatant became clear. The red blood cells were then resuspended in saline to prepare a working suspension. Aliquots of the RBC suspension were incubated with the test samples at 37 °C for 30 min. Following incubation, the samples were centrifuged, and the optical density of the supernatant was recorded at 540 nm.

### 4.11. Analysis of the Radical Scavenging Capacity of Hydrogels

The antioxidative capacity of the hydrogel samples was determined by an ABTS^·+^ radical scavenging assay, which was conducted following an improved method reported in a previous study [47].

### 4.12. Measurement of Intracellular ROS Levels

Intracellular ROS levels were evaluated using the DCFH-DA fluorescent probe (servicebio, China) [48]. Briefly, HUVECs and RAW264.7 cells were seeded into 24-well plates and allowed to adhere overnight. Following attachment, the cells were exposed to H_2_O_2_ or LPS alone or in combination with hydrogel treatments. Afterward, the cells were rinsed with PBS and incubated with DCFH-DA in serum-free medium at 37 °C for 30 min in the dark. Subsequently, the unbound probe was removed by extensive washing, and DCF fluorescence (green), reflecting ROS production, was visualized and recorded under a confocal microscope.

### 4.13. Cell Scratch Assay

Cell migration capacity was assessed using a scratch assay. The brief procedure is as follows: HUVECs were cultured in 12-well plates and left to attach overnight prior to further treatment. Following oxidative damage induction with H_2_O_2_ (200 μM) for 2 h, scratches were created using sterile pipettes. After removal of the old medium, hydrogel extract was added for continued cell maintenance. Photographs of the same field of view were taken at 0, 12, and 24 h under an inverted microscope. Quantitative analysis of scratch closure area was performed using ImageJ 1.52a software to evaluate cell migration.

### 4.14. Enzyme-Linked Immunosorbent Assay

The concentrations of TNF-α and IL-6 in the supernatant were determined using commercially available ELISA kits (liankebio, Hangzhou, China), with all procedures performed in accordance with the manufacturer’s instructions.

### 4.15. Western Blot Analysis

Protein expression was assessed via Western Blot analysis [49]. In brief, we seeded RAW 264.7 cells into 6-well plates and then exposed them to LPS (200 ng/mL) and test samples (such as drug-containing medium or hydrogel extracts) for 24 h. Subsequently, the cells were harvested in RIPA lysis buffer containing protease and phosphatase inhibitors. Protein concentration was determined using the Bicinchoninic Acid method. Equivalent quantities of protein were subjected to SDS-PAGE and subsequently transferred to PVDF membranes. Following a blocking step, the membranes were exposed to primary antibodies overnight at 4 °C, after which they were incubated with HRP-conjugated secondary antibodies for 1–2 h at room temperature. Finally, visualisation was achieved using ECL chemiluminescent substrate (Yeasen Biotechnology, Shanghai, China), with quantitative analysis of band intensity performed via grey-scale analysis using ImageJ software.

### 4.16. Establishment of Diabetic Full-Thickness Skin Wound Model and Treatment

Diabetes was induced in male C57BL/6 mice by intraperitoneal injection of streptozotocin (STZ) at 50 mg/kg for five consecutive days [50,51]. Fasting blood glucose levels were measured via tail vein sampling 24 and 72 h after the final injection. Mice exhibiting persistently elevated fasting blood glucose above 11.1 mmol/L alongside classic symptoms such as polydipsia, polyuria, and weight loss were deemed successful diabetes models. Following successful modelling, mice were anaesthetised, with the dorsal skin shaved and disinfected. A circular full-thickness skin defect approximately 8 mm in diameter was created using surgical scissors. Subsequently, the mice were randomly divided into a control group, a model group, an FQ group, an OSSP group, an OSSP@FQ group, and an OSSP@FQ&GOX group, with 9 mice in each group. and were given the treatment once a day. Apply 20 μL of FQ micelles or hydrogel to the skin wound once a day. Photographs of the wound were taken at regular intervals to document healing progression, and wound closure rates were calculated.

### 4.17. Histological Analysis

The collected tissue samples underwent fixation, embedding, and sectioning in sequence [51]. H&E staining, Masson’s trichrome staining, and immunofluorescence assays were performed according to the respective protocols.

### 4.18. Statistical Analysis

GraphPad Prism 10.1.2 and Origin 2021 were employed for all statistical analyses. All data were expressed as mean ± SD. Statistical significance was determined using the following thresholds: *p* > 0.05, not significant (ns); *p* < 0.05, *; *p* < 0.01, **; and *p* < 0.001, ***.

## Data Availability

Data available on request from the authors.

## Supplementary Materials

The following supporting information can be downloaded at: https://www.mdpi.com/article/10.3390/gels12070652/s1.
