# Supplementary material for: Enzyme-Flavonoid Synergistic Hydrogel: Enables Glucose-Activated Cascade Acidification and Programmed Drug Release for Diabetic Wound Therapy
Source: Gels. 2026 Jul 21;12(7):652. doi: 10.3390/gels12070652 (PMC13407840; doi:10.3390/gels12070652)
Supplement: Supplementary file 1 [file gels-12-00652-s001.zip › gels-4390526-supplementary.pdf]

Supporting Information

Enzyme-Flavonoid Synergistic Hydrogel: Enables Glucose-Activated Cascade Acidification and Programmed Drug Release for DiabeticWound Therapy

Guixi Wang [1,†], Sihang Shen [2,†], Yichen Tian [2], Chao Li [2], Junnan He [3,*] and Yuzhu Song [2,*]

1 School of Basic Medical Sciences, Kunming Medical University, Kunming 650500, China

2 Research Center of Molecular Medicine of Yunnan Province, Faculty of Life Science and Technology, Kunming University of Science and Technology, Kunming 650500, China

3 Yunnan Key Laboratory of Modern Separation Analysis and Substance Transformation, College of Chemistry and Chemical Engineering, Yunnan Normal University, Kunming 650500, China

* Correspondence: hejunnan@ynnu.edu.cn (J.H.); yuzhusong@kmust.edu.cn (Y.S.)

† These authors contributed equally to this work.

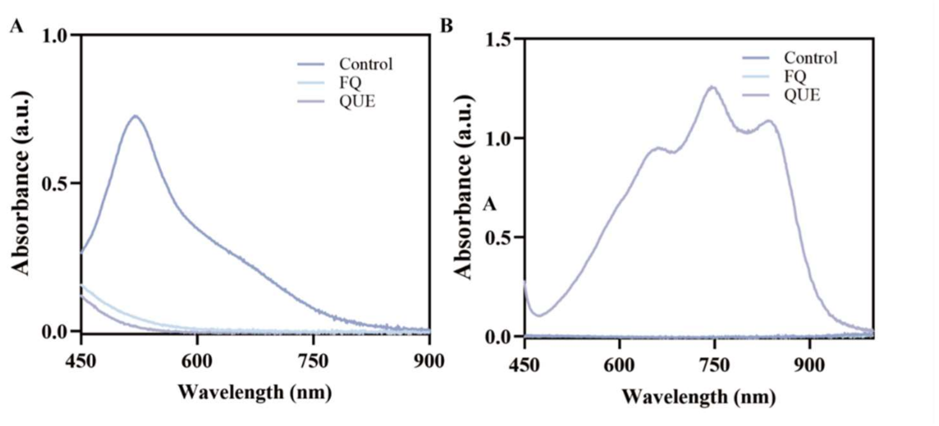

Figure S1. Scavenging capacity of FQ micelles towards (A) DPPH and (B) ABTS radicals.

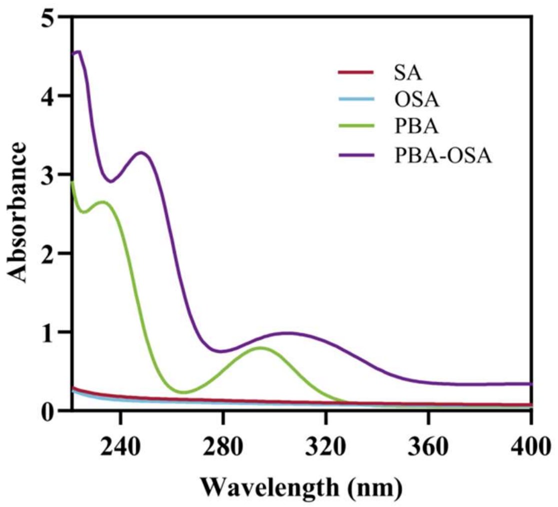

FigureS2. UV-visible absorption spectroscopy of PBA-OSA.

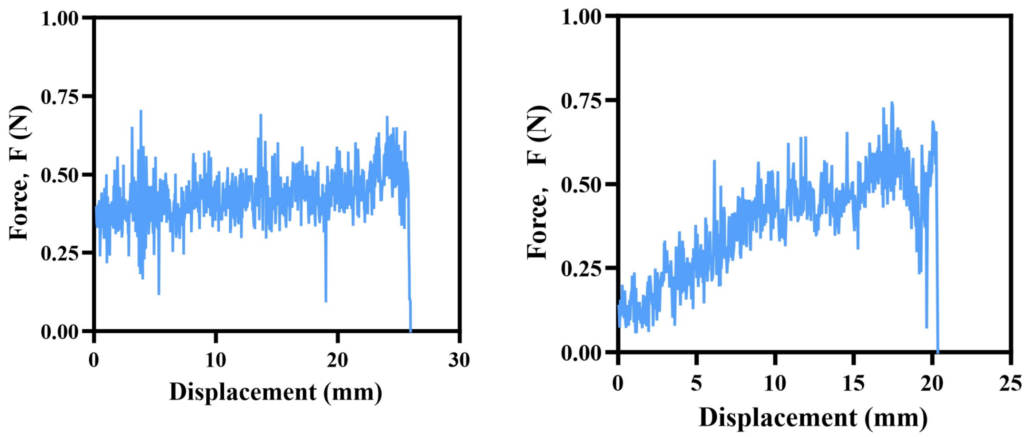

FigureS3. Lap-shear strength measurements of OSSP and OSSP@FQ&GOX hydrogels.

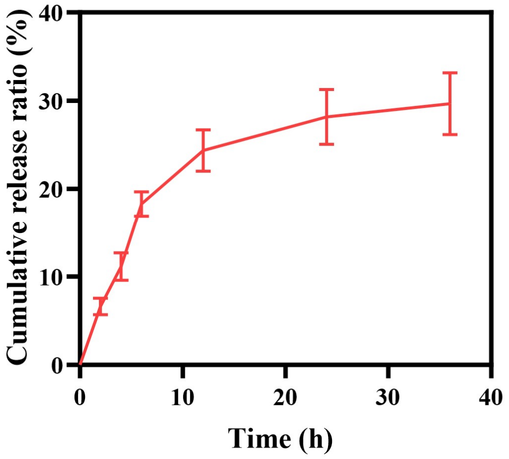

FigureS4. Cumulative release rate of glucose oxidase at pH 7.4.

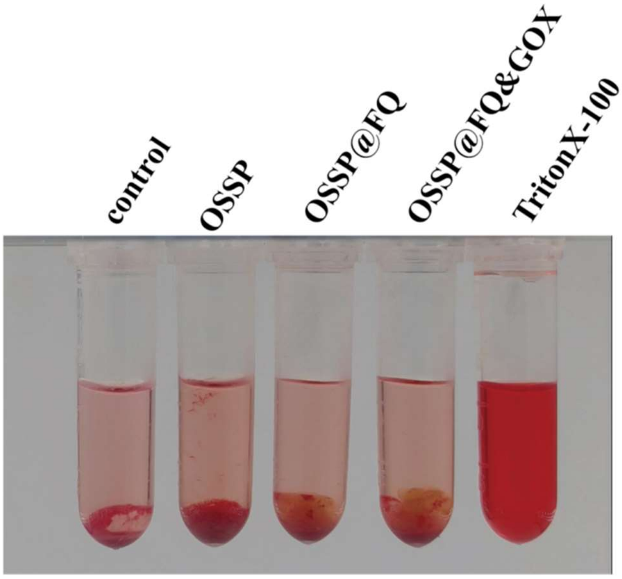

Figure S5. Image demonstrating the haemolytic activity of the hydrogel.

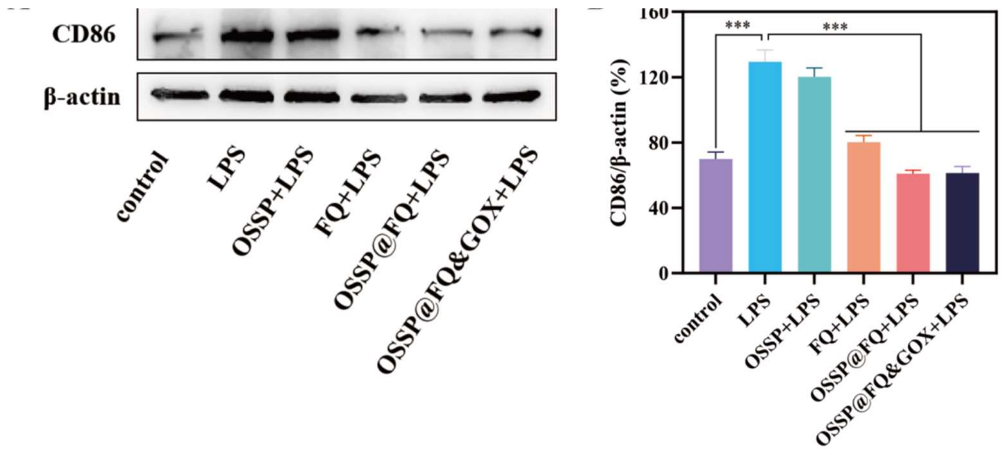

Figure S6. Western blot analysis for (A) the expression and (B) relative quantification of CD86 in RAW264.7 cells.

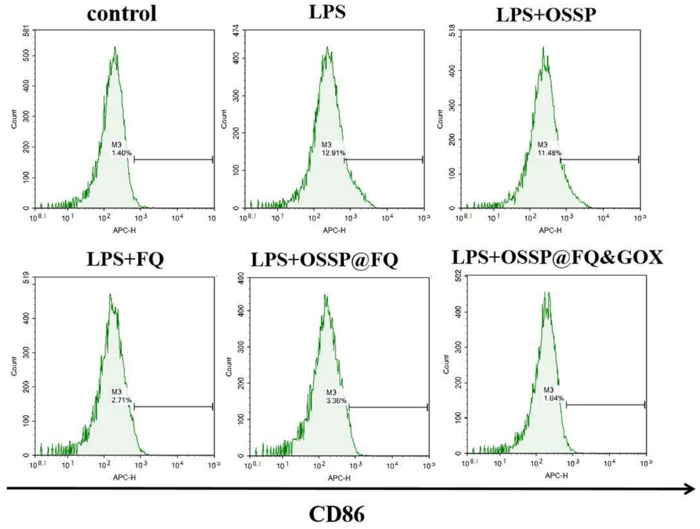

Figure S7. Flow cytometric analysis of CD86 expression on macrophages.

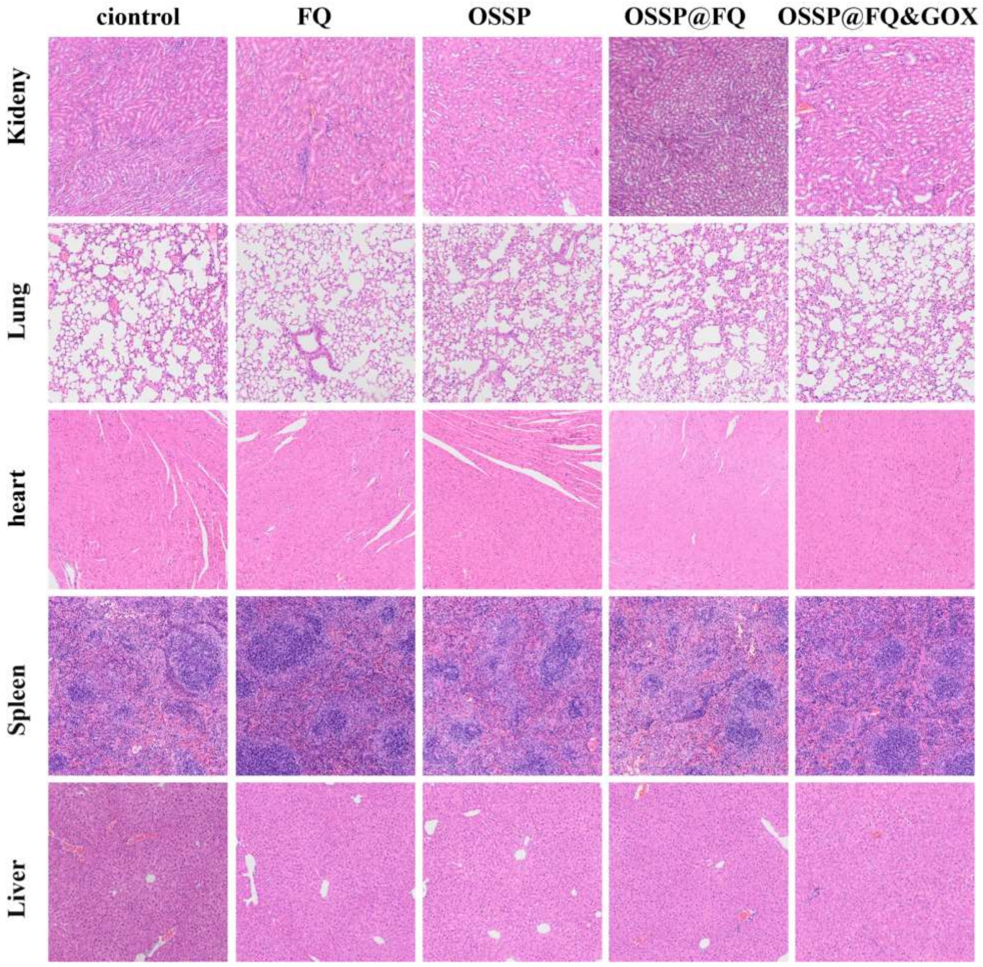

Figure S8. Toxicity evaluation of the hydrogel in animals, observing the morphological changes of the heart, liver, spleen, lung, and kidney organs through H&E staining.

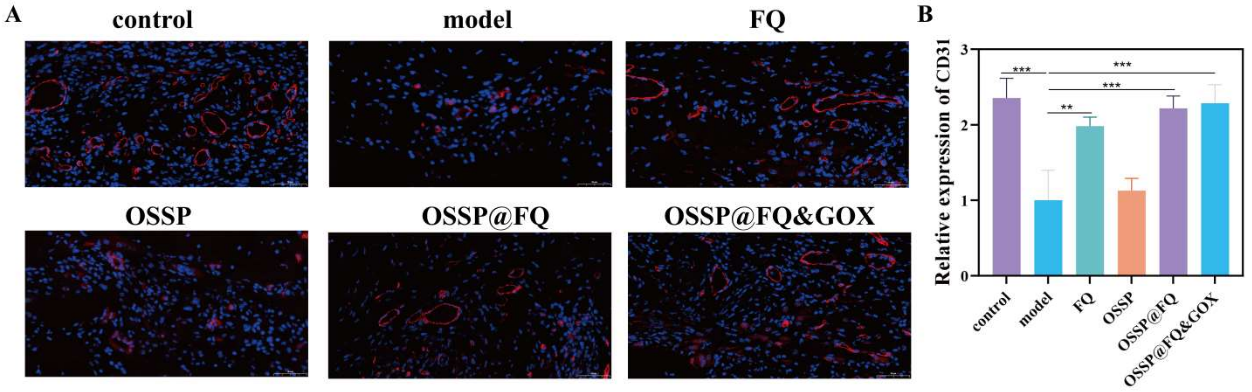

Figure S9. (A) Representative immunofluorescence staining images of CD31; (B) Quantitative analysis of relative fluorescence intensity for CD31.
